# Exodin, a New Purgative1From the Göttingen University Medical Clinic; read before the Göttingen Medical Society, December 3, 1903; abstracted from the *Deutsche Medizinische Wochenschrift*, January 1, 1904.

**Published:** 1904-03

**Authors:** Wilhelm Ebstein

**Affiliations:** Göttingen


### ABSTRACTS.

### Exodin, a New Purgative.[1]

By PROF. WILHELM EBSTEIN, Göttingen.

EXODIN, the diacetylrufigallic-acid-tetramethyl-ether, is a yellow powder melting at 180 to 190° C. It is odorless and tasteless, insoluble in water and with difficulty dissolved in alcohol. Since January, 1903, I have experimented with it very extensively in the University and in my private clinic as well as in consulting practice. This paper is based upon numerous histories of more or less obstinate constipations in males and females of all ages.

The remedy never causes unpleasant by-effects, such as dyspeptic symptoms or eructations. It usually acts in from 8 to 12 hours. The unpleasant diarrheal discharges produced by many otherwise effective purgatives are absent with exodin. In most cases the first passages are mushy and even solid. In the course of the next few hours there are usually from one to three more passages, the last not infrequently being thin. The feces preserve their natural color. In extremely rare cases the first evacuations are diarrheal; entirely watery discharges were hardly ever observed. Once in a while the remedy may be ineffectual, but this is wholly exceptional.

The action of exodin is midway between laxative and purgative. It can be used as an evacuant in simple constipation in otherwise healthy persons and also when sluggishness of the bowels is an accidental complication or a part of some other affection. In pregnancy, even during the first two months, exodin has, when all other laxatives failed, proved very efficacious and harmless, causing evacuation of abundant stools without any trouble. The use of the drug at intervals showed that it is always equally active whenever readministered.

For obvious reasons, a universal purgative suitable for all fecal retentions, will probably never be discovered. But the properties which exodin exhibits enables me to welcome it as a valuable purgative. To find the indications for constipation remedies requires especially a careful abdominal examination. In my book on Chronic Constipation I have gone fully into all the points involved. There is too much routine prescribing done here; those who are perpetually talking and writing against quackery must not forget the importance of depriving the quacks of their weapons.

---

1. From the Göttingen University Medical Clinic; read before the Göttingen Medical Society, December 3, 1903; abstracted from the *Deutsche Medizinische Wochenschrift*, January 1, 1904.

Too much must not be demanded of exodin. We cannot expect the intestines to react at once to the purgative in cases of old chronic coprostasis, which is kept up by hard and dried scybalæ accumulated in the large intestines; or in spastic constipations in which the spasm of the intestinal muscles prevents defecation; or where paralytic conditions cause the retention. When the intestinal canal is weakened, its refusal to perform its functions becomes more and more obstinate as we irritate it, either by large doses of mild purgative or by the always reprehensible method of employing so-called drastic evacuants. When hardened fecal masses are accumulated in the large bowel and purgatives are ineffectual, the so-called high oil enemata are indicated. But the acid contents of the oil, increased though it is in the gut, is not infrequently unable to irritate the large intestine sufficiently to cause the expulsion of the feces. To aid in this, I employ purgatives which I call "propellers," and which are extremely important in the treatment of chronic coprostasis. A proper combination of the oil with them must be made, care being taken to strengthen and not to weaken the intestinal action. In my book already referred to I gave a list of the propellers at that time known to me.

The result of very extensive experimentation with Boudard's recinine pills has caused me to reject them. But exodin has done me excellent service as a "propeller"; since I began using it for that purpose I have completely given up calomel and castor oil. Frequently 15-grain doses of the remedy have sufficed, with oil enemata, to secure satisfactory evacuations.

Over purgatin and emodin (see my report on both remedies in the *Therapie d. Gegenwart,* Jan., 1902), exodin has the advantage of greater efficiency, results being obtained with smaller doses. Besides, it does not make the urine stain, to which women, for readily understood reasons, so strongly object. Patients suffering from the severest forms of chronic constipation who had tried all possible remedies and whose judgment was unimpeachable, preferred exodin on account of its prompt, painless and efficient action to all other medicaments.

I made an extensive series of experiments with exodin, adding it to the oil enemata, but the administration by mouth seemed to act better. Exodin is marketed in the form of 7½-grain tablets, which are tasteless and odorless. One tablet is enough for children. Adults take from one to three tablets; two, however, are usually sufficient to produce one or several mushy stools within 8 to 12 hours. The tablets should be allowed to disintegrate in a suitable quantity of water and the mixture drunk under constant stirring with a spoon. Any exodin remaining in the glass should be rinsed down with additional water. This method insures the introduction of the remedy into the stomach in the finest possible state of subdivision.

